# How Accurate Is Preoperative Evaluation of Pelvic Organ Prolapse in Women Undergoing Vaginal Reconstruction Surgery?

**DOI:** 10.1371/journal.pone.0047027

**Published:** 2012-10-09

**Authors:** Haim Krissi, Ram Eitan, Edward Ram, Yoav Peled

**Affiliations:** 1 Urogynecology Unit, Department of Obstetrics and Gynecology, Helen Schneider Hospital for Women, Rabin Medical Center, Petach Tikva, affiliated with Sackler Faculty of Medicine, Tel Aviv University, Tel Aviv, Israel; 2 Division of Surgery, Hasharon Hospital, Rabin Medical Center, Petach Tikva, affiliated with Sackler Faculty of Medicine, Tel Aviv University, Tel Aviv, Israel; Tehran University of Medical Sciences, Islamic Republic of Iran

## Abstract

**Objective:**

To evaluate the differences between the in-office and intraoperative techniques used to evaluate pelvic organ prolapse.

**Materials and Methods:**

A prospective study included 25 women undergoing vaginal reconstruction surgery including vaginal hysterectomy for pelvic organ prolapse. The outpatient pelvic and site-specific vaginal examination was performed in the lithotomy position with the Valsalva maneuver. Repeated intraoperative examination was performed under general anesthesia with standard mild cervical traction. The Pelvic Organ Prolapse Quantification system (POPQ) was used for both measurements and staging. The values found under the two conditions were compared.

**Results:**

The intraoperative POPQ-measurements values were significantly higher than the outpatient values for apical wall prolapse in 17/25 (68%) women and for anterior wall prolapse in 8/25 (32%) women. There was not a significant difference in the posterior wall where increase in staging was shown in 3/25 (12%) patients.

**Conclusions:**

Clinicians and patients should be alert to the possibility that pelvic organ measurements performed under general anesthesia with mild traction may be different from preoperative evaluation.

## Introduction

Pelvic organ prolapse is a common indication for vaginal gynecologic surgery [1]. Women have an 11% life-time risk of surgery for pelvic organ prolapse or urinary incontinence [2]. The use of imprecise or inconsistent classifications of pelvic organ prolapse by clinicians may impair clinical communication, patient follow-up, and meaningful comparisons between studies. To avoid these limitations, the International Continence Society introduced the Pelvic Organ Prolapse Quantification system (POPQ) as the standard tool to quantify, describe, and stage uterovaginal prolapse [3].

The quantification procedure in patients with pelvic organ prolapse is usually performed on an outpatient basis in the dorsal lithotomy position using a forceful Valsalva maneuver. However, the final vaginal examination is usually performed intraoperatively under general anesthesia after mild traction of the cervix in the horizontal axis. These differences in technique may lead to different findings and, as a consequence, a need to change the surgical approach.

The objective of the present study was to find the difference between the preoperative and intraoperative evaluation of pelvic organ prolapse.

**Table 1:** Clinical and demographic characteristics for the study group.

Clinical characteristics	Mean±SD (range)
Age (years)	63.4±10.2 (44–79)
Parity (average)	2.9±0.9 (2–5)
BMI (kg/m^2^)	22.5±4.3 (19–38)
Weight of largest baby (gr.)	3534±402 (2950–4210)
Duration of prolapse (month)	34±25 (1–120)
**Uterine Parameters**	
Cervical length (cm)	6.7±1.7 (4–11)
Cervical width (cm)	2.4±0.5 (2–4)
Uterine length (cm)	5.2±1.8 (3–10)
Uterine width (cm)	4.2±1.5 (3–8)

BMI – body mass index.

## Materials and Methods

A prospective study was conducted at a tertiary university-affiliated medical center. The study population consisted of 25 consecutive women with combined prolapse of the apical, anterior, and posterior compartments. All women were scheduled for vaginal hysterectomy with anterior and posterior colporrhaphy without meshes under general anesthesia. Women with a uterine size greater than 12 weeks’ gestation by vaginal examination, fourth degree uterine prolapse, previous vaginal or abdominal pelvic surgery, or collagen or neurological disease and spinal anesthesia were excluded from the study. All participants provided written informed consent, and the study protocol was approved by the local institutional review board.

The preoperative office examination was performed by the same physician in all cases and included a detailed pelvic examination and site-specific vaginal examination in the lithotomy position; a Sims speculum was used during the Valsalva maneuver for proper visualization. Patients were encouraged to perform the maximal Valsalva maneuver and to cough. The patients were asked to confirm that the prolapse resembled what they experienced during daily activities. Pelvic support in all compartments was scored according to the POPQ [3].

All patients underwent a preoperative multichannel urodynamic evaluation with prolapse reduction to identify overt or occult stress incontinence. When the vaginal defect was combined with stress incontinence, additional continence surgery was performed.

Prior to surgery, an enema was administered to empty the bowel. After the induction of general anesthesia, the patients were paralyzed and ventilated, placed in the lithotomy position, the vagina was cleaned with polidine solution, and the surgical area was covered in a sterile manner. The surgeon then grasped the uterine cervix with a Braun tenaculum forceps (KLS Martin Tuttlingen, Germany) attached to a standardized scaled metal coil (Yavin Yeda, Caesarea, Israel) and pulled it horizontally very gently at a constant force of 0.5 kg (500 Newton) for standardization while repeating the POPQ measurements. This technique for vaginal hysterectomy is considered by some as the standard first step to facilitate the procedure [4]–[6]. The traction force used was based on previous reports on passive support of the uterus [7], [8]. The preoperative data collection was performed in the preoperative evaluation where the intraoperative measurement performed blindly to the preoperative measurement.

All measurements were recorded on an electronic data sheet, and analyzed using SPSS software for Windows version 17. The values of the POPQ assessments made on an outpatient and intraoperative basis were compared.

The Wilcoxon signed-rank test was applied to the paired observations to analyze differences between the preoperative and intraoperative POP-Q points and chi square test was performed for the proportion of patients in each stage before and after anesthesia. A sample size calculation analysis, assuming a power of 80% and a significance value of 5%, showed that 17 patients in each arm (before and after anesthesia) are needed, assuming a mean apical stage of 2.0 and a standard deviation of 0.6, for a difference of 0.6. A p value of less than 0.05 was considered significant.

## Results

Twenty-five patients were included in the study. Table 1 describes their demographic and clinical characteristics. Tables 2 show the POP-Q measurements preoperatively and postoperatively. The mean apical and anterior vaginal wall descent was significantly greater on the intraoperative than the outpatient assessment, with no significant difference in posterior wall measurements.

**Table 2:** POPQ measurements with the Valsalva maneuver preoperatively and under general anesthesia with uterine traction intraoperatively.

Points	Preoperative	Intraoperative	P value
Aa	1.1±1.7	1.5±1.4	0.039
Ba	1.2±1.6	1.6±1.4	0.008
C	0.2±2.3	2.7±2.2	<0.001
Ap	−1.3±1	−1.3±1	NS
Bp	1.2±1	1.2±1	NS
D	−4.2±2.1	−3.4±2.5	0.004
**Genital hiatus (cm)**		4.4±1.1 (range 3–7)	
**Perineal body (cm)**		1.1±0.5 (range 0.5–2)	
**Total vaginal length (cm)**		8.5±0.9 (range 7–10)	

All values expressed in mean±standard deviation.

Aa – anterior vaginal wall 3 cm proximal to the hymen.

Ba – most distal position of the remaining upper anterior vaginal wall.

C – most distal edge of the cervix or vaginal cuff.

D – posterior fornix.

Ap – posterior vaginal wall 3 cm proximal to the hymen.

Bp – most distal position of the remaining upper posterior vaginal wall.

Staging analysis (Table 3) showed that intraoperative stage was higher than preoperative values in 17/25 women (68%) for apical prolapse, in 8/25 for anterior wall prolapse (32%) and in 3/25 patients (12%) for posterior wall prolapse. The only statistically significant increase in intraoperative stage was in apical prolapse stage 1 (p = 0.002). Since all women had a combined apical, anterior, and posterior wall defect preoperatively, these findings did not warrant a change in the surgical plan. No patient had a lesser degree of prolapse intraoperatively.

**Table 3:** Stage of prolapse with Valsalva maneuver preoperatively and under general anesthesia with uterine traction intraoperatively.

Site of prolapse	Stage	PreoperativeNo(%)	IntraoperativeNo(%)	P value
Apical prolapse	1	8(32)	0	0.002
	2	10(40)	11(44)	0.774
	3	7(28)	12(48)	0.145
	4	0	2(8)	0.149
Anterior vaginal wall	1	6(24)	3(12)	0.463
	2	7(28)	7(28)	1.0
	3	12(48)	14(56)	0.778
	4	0	2(8)	0.490
Posterior vaginal wall	1	20(80)	18(72)	0.742
	2	4(16)	5(20)	0.886
	3	1(4)	2(8)	0.809
	4	0	0	–

Stage 0 no prolapse.

Stage 1 stage 0 criteria not met + leading edge < −1 cm.

Stage 2 leading edge ≥ −1 cm but ≤ +1 cm.

Stage 3 leading edge +1 cm but < +(tvl −2) cm.

Stage 4 leading edge ≥ +(tvl −2) cm.

Leading edge, cervix – uterine (apical) prolapse.

Leading edge, anterior – anterior wall prolapse.

Leading edge, posterior – posterior wall prolapse.

Vaginal hysterectomy including lateral fascia repair of the anterior and posterior walls was performed in all patients. In addition, 8 patients underwent an anti-incontinence procedure consisting of tension-free vaginal tape in 3 and trans-obturator tension-free vaginal tape in 5.

## Discussion

The results of the present study demonstrate that preoperative office POPQ-based measurements and staging of apical, anterior, and posterior vaginal wall prolapse, performed in the lithotomy position with the Valsalva maneuver, do not match the values derived intraoperatively. Significantly greater apical and anterior wall prolapse were found under general anesthesia with gentle traction.

During targeted physical examination of prolapse, it is critical that the examiner sees and describes the maximum protrusion experienced by the individual during her daily activities. There is no objective scientific method to induce maximal prolapse prior to surgery. Accepted in-office method is the use of Valsalva maneuver with patient confirmation of maximal prolapse. This is preferable to another in-office practice wherein the surgeon held the tenaculum and pulled on it while the patient coughed to induce maximal uterus descent. This procedure is painful to the patient and may yield less accurate results.

In standard practice, although we cannot guarantee that the preoperative physical assessment will yield comparable results to the intraoperative assessment, it nevertheless serves as the basis for the surgical plan and the explanation provided to the patients. In our study, the increased descent of the uterus and vaginal walls intraoperatively may be related not only to the traction but also to the relaxation of the tissue under general anesthesia.

Vineyard et al. [7], using the Baden-Walker system in selected cases, found greater prolapse at surgery than preoperatively in up to 32% of patients. Additionally, Vierhout et al. [8] reported more pronounced prolapse in the middle and posterior compartments at surgery than preoperatively although their sample group was heterogeneous.

Our results are in line with the anatomic relationship between the uterus and the anterior vaginal wall compared to the posterior vaginal wall. The bladder lies on the anterior vaginal wall, in close proximity to the uterus; therefore, as uterine prolapse increases, anterior wall prolapse increases as well. However, the rectum lies beneath the posterior vaginal wall and has no connection to the uterus, so posterior wall prolapse is not significantly affected by uterine descent.

Although the size of our patient sample was small, the study was designed to minimize the confounding surgical factors including type of surgery, sites of prolapse, and mode of anesthesia. The study group was limited to women with combined prolapse of the three compartments. In women without all compartment prolapse, different intraoperative findings may lead to changes in the planed surgery.

The increased stage of prolapse is of less significance for an experienced surgeon. However, increase in apical prolapse may facilitate the hysterectomy.

The preoperative examination performed without bowl empting while the intraoperative examination performed after an enema. The influence of enema on prolapse examination is unknown. However, the posterior wall prolapse was not significantly changed intraoperatively.

This study is an alert for surgeons and patients to the possibility that the degree of prolapse determined in the office may differ when reassessed under general anesthesia and traction. Patients should be informed that the surgical plan may need to be altered according to the intraoperative findings.
